# A Green Approach for the Synthesis of Silver Nanoparticle-Embedded Chitosan Bionanocomposite as a Potential Device for the Sustained Release of the Itraconazole Drug and Its Antibacterial Characteristics

**DOI:** 10.3390/polym14091911

**Published:** 2022-05-07

**Authors:** Saruchi, Manpreet Kaur, Vaneet Kumar, Ayman A. Ghfar, Sadanand Pandey

**Affiliations:** 1 Department of Biotechnology, CT Institute of Pharmaceutical Sciences, Shahpur Campus, Jalandhar 144008, Punjab, India; manndhillon1993@gmail.com; 2 Department of Applied Sciences, CT Institute of Engineering, Management and Technology, Shahpur Campus, Jalandhar 144623, Punjab, India; vaneet2106@gmail.com; 3 Department of Chemistry, College of Science, King Saud University, P.O. Box 2455, Riyadh 11451, Saudi Arabia; aghafr@ksu.edu.sa; 4 Department of Chemistry, College of Natural Sciences, Yeungnam University, 280 Daehak-Ro, Gyeongsan 38541, Gyeongbuk, Korea

**Keywords:** *Saccharum officinarum*, silver nanoparticles, chitosan, bionanocomposite, antibacterial activity

## Abstract

The present research work intended to demonstrate the green synthesis of silver nanoparticles (AgNPs) using the plant extract *Saccharum officinarum*, and then the development of chitosan–silver (CH-Ag) bionanocomposite. The synthesized AgNPs were characterized using UV spectroscopy, Fourier transform infrared (FTIR), and transmission electron microscopy (TEM). The maximum absorption spectrum peak was observed at 420 nm, revealing the formation of AgNPs by the stem extract of *S. officinarum*. The AgNPs sizes were in the range of 10–50 nm. Itraconazole is an antifungal that is used as a novel drug to study its release through synthesized bionanocomposite. Different kinetic models, such as zero order, first order, Korsmeyer–Peppas, Hixson–Crowell and Higuchi, were used to study the drug release profile from the synthesized CH-Ag bionanocomposite. The first-order kinetic model showed the best fit for the drug release with the maximum regression coefficient value. The antibacterial activity of the synthesized CH-Ag bionanocomposite was examined against *Bacillus cereus*, *Staphylococcus*, and *Escherichia coli*, and it was shown to be efficient against these strains.

## 1. Introduction

The recent years have observed remarkable growth in research and applications in the areas of nanotechnology and nanoscience. Nanotechnology has been utilized in different fields, such as environmental, health care, chemical industries, photo-electrochemical applications, etc. Nanobiotechnology provides methods for the nontoxic and environmentally friendly synthesis of metal nanoparticles [1,2,3]. Silver nanoparticles (AgNPs) possess numerous anti-inflammatory and anti-bacterial properties that facilitate quick wound healing. Additionally, silver nanoparticles can be used for wound dressings, medical implants, pharmaceutical preparations, and biomedical applications. Nanoparticles possess a high surface area-to-volume ratio, making them excellent catalysts [4,5,6,7].

Globally, sugarcane is the leading sugar crop. In addition to liquids, syrups, and capsules, sugarcane is essential for preserving various kinds of medicine. It is an excellent source of energy drinks [8,9,10]. Triazoles are the first-line managers for the preclusion and cure of persistent and allergic fungal infections. Itraconazole (cis-4[4-4-4[[2-(2-4-dichlorophenyl)-2-(1H-1,2,4,triazol-1-methyl)-1,3-dioxolan-4-yl]-1-piperazinyl]phenyl]-2,4-dihydro-2-(1-methyl-propyl)-3H-1,2,4-triazol-3-one) was initially synthesized in 1980, and has broad-spectrum antifungal activity (especially in filamentous fungi, such as *Aspergillus* spp.), potency and toxicity profiles, because of the presence of the triazole ring in the side chain, which makes it very active. It acts as an anti-allergic agent, and is used for the treatment of aspergillosis, candidiasis, dermatophyte infections, sporotrichosis, blastomycosis, histoplasmosis, etc. [11,12,13]. Chitosan is a natural polysaccharide, which is a cationic copolymer and is hydrophilic in nature. It is biocompatible, as well as biodegradable, and for that reason it is widely used in the pharmaceutical and food industries [14].

This study targets the green synthesis of AgNPs using the plant extract *Saccharum officinarum* and the synthesis of chitosan–Ag-based bionanocomposites. The antifungal drug itraconazole was released under controlled conditions using CH-Ag nanocomposites. A number of bacterial strains were also tested for the antibacterial activity of the CH-Ag bionanocomposite, including *Bacillus cereus*, *Staphylococcus*, and *Escherichia coli.*

## 2. Materials and Methods

### 2.1. Materials

Sugarcane was taken from Nakodar co-operative sugar mill (NCSM), Gaggarwal Village, Mehatpur Road, Nakodar, Jalandhar, Punjab, India Silver nitrate (AgNO_3_) (AVARICE, ISO 9001:2015, Ghaziabad, India) and chitosan (C_6_H_11_NO_4_)n (AVARICE, ISO 9001:2015, Ghaziabad, India) were used as received; itraconazole was gifted by the pharmaceutical industry (Cipla Pvt. Ltd. Baddi, India). Erythromycin and ampicillin were purchased from (MP Biomedia, Maharashtra, India).

### 2.2. Methods

#### 2.2.1. Preparation of Sugarcane Extract

**Figure 1:**
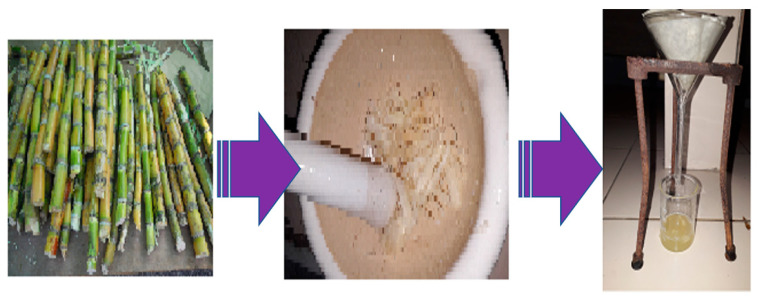
Pictorial presentation of extraction of sugarcane extract from sugarcane.

A small sugarcane stem was cut into slices and washed properly in distilled water. After that, 10.0 g of small sugarcane slices were crushed properly in the pestle and mortar. The extracted organic material was filtered on Whatman no. 1 filter paper (Figure 1).

#### 2.2.2. Green Synthesis of AgNPs

**Figure 2:**
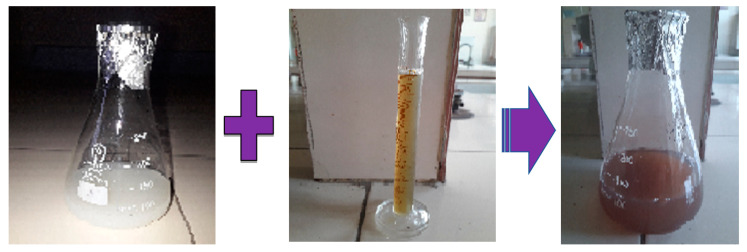
Synthesis of AgNPs using sugarcane extract.

The synthesis of AgNPs was achieved by putting 90 mL of a 1 mM silver nitrate solution in a reaction flask and stirring it with 10 mL of sugarcane extract. During incubation, the reaction mixture was maintained at 37 °C. After the sugarcane extract was added, the solution changed color from pale yellow to brown. This is a clear indication that silver nanoparticles were forming (Figure 2) [15,16,17,18].

#### 2.2.3. Synthesis of AgNP-Embedded Chitosan (CH-Ag) Bionanocomposite

AgNP-embedded chitosan (CH-Ag) bionanocomposite was prepared by placing 1g of chitosan in a reaction flask. To this, 0.5 mL of acetic acid was added, followed by the addition of 50 mL of distilled water. This dissolved the chitosan powder properly in the reaction flask. Then, 1.0 mM of silver nitrate was added to the reaction flask while stirring using a magnetic stirrer. The formed suspension was kept for 2 h and then 10 mL of *S. officinarum* extract was added. In the present study, sugarcane was used as a stabilizing and reducing agent. The main function of using the reducing agent for the synthesis of NPs is to reduce what is present in the metal salts to atoms. In the sugarcane leaf extract, there are phenolic compounds present, which act as reducing agents for the synthesis of AgNPs from its precursor. After the addition of *S. officinarum* stem extract, the color of the reaction solution changed from pale yellow to brown, indicating the formation of AgNPs in the suspension (Figure 2). The formed reaction solution was then centrifuged at 10,000 rpm for 10 min. The attained filtrate was washed with double distilled water. The formed pellets were dried in a hot air oven and used for advanced studies [19,20,21].

### 2.3. Characterization of AgNPs

The synthesized AgNPs were analyzed by a UV–Vis spectrophotometer (MODEL LT-29). Transmission electron microscopy (TEM) utilizes energetic electrons, which provide the morphological nature of AgNPs. TEM was performed at Punjab Agriculture University, Ludhiana, Punjab, India. Fourier transform infrared (FTIR) analysis of the synthesized materials was performed at NIT Jalandhar, Punjab, India.

### 2.4. Drug Loading

The drug carrier device should have a high drug-loading capacity, so that a lesser quantity of matrix materials is required for administration. There are two methods for drug loading:

- Incorporation of the drug at the time of nanoparticle synthesis;
- Imbibing the drug after the formation of nanoparticles.

In the present work, the itraconazole drug was loaded in synthesized bionanocomposite using the swelling–diffusion method. Synthesized bionanocomposite (0.5 g) was immersed in the drug solution (0.2 mg/mL). It was incubated at 37 °C for 48 h. After 48 h, drug-loaded bionanocomposites were taken out and washed with double distilled water to remove any surface-adhered drug particles. After washing, the sample was kept in a hot air oven at 45 °C until it was dried. The drug release behavior was studied with the help of a double-beam UV–VIS spectrophotometer. Drug entrapment efficiency (*EE*) was calculated using the following equation [22,23,24,25]:

(1) $$EE\left(\%\right)=\left(\frac{W_{f}}{W_{o}}\right)\times 100$$

In this equation, *W_f_* and *W_o_* are the total amount of drug in the solution before loading and after loading, respectively.

### 2.5. In Vitro Drug Release Studies

Phosphate buffer solution was prepared in distilled water using di-sodium hydrogen phosphate and sodium di-hydrogen phosphate, and the pH of the solution was maintained at 2, 7 and 9.2 with the help of 1 M NaOH. Drug release was studied at pH 2, 7 and 9.2, which corresponds with the pH of gastric fluid, blood stream and intestinal fluid, respectively. Drug release through CH-Ag bionanocomposite was deliberated by immersing the drug-loaded bionanocomposite in different pH solutions at a temperature of 37 °C. Drug release concentration in the solution was studied every one hour using a double-beam UV–VIS spectrophotometer [26,27,28,29].

### 2.6. Mechanism of Drug Release

Drug release behavior of the synthesized bionanocomposite was studied using the power law equation, which is expressed as [30,31,32,33]:

(2) $$\frac{M_{t}}{M_{\infty }}=kt^{n}$$

where $M_{t}$/*M*_∞_ is the drug release at time *t*, and *k* and *n* are the rate constant and diffusion exponent of drug release, respectively. The *n* value showed the type of drug release mechanism. The drug release experiments were conducted in triplicate, so as to avoid any error. Drug diffusion mechanism is explained by Fick’s first and second laws, as follow:

*M_s_* = *kt^n^* (3)

where *k* and *n* are gel characteristic constant and diffusion exponent, respectively. Further, *n* = 0.5, 0.5 to 1, and 1 or >1 signify a Fickian, non-Fickian, and case II diffusion mechanism, respectively. Fick’s power law equation was used to calculate the drug release via synthesized CH-Ag bionanocomposite.

*M_t_*/*M*_∞_ = *kt^n^* (4)

where *M_t_* and *M*_∞_ are the fractional release of the drug at different time intervals and at equilibrium, respectively. The value of n and k are obtained from the slope and intercept of the plot between *M_t_*/*M*_∞_ and lnt.

Initial diffusion coefficient (*D_i_*) was calculated using the following equation:

*M_t_*/*M*_∞_ = 4 × *D_i_*/*πl*^2^ (5)

where *l* is the thickness of the IPN sample used.

Average diffusion (*D_A_*) was calculated from the following equation:

*D_A_* = 0.049l^2^/t^1/2^ (6)

where t_1/2_ is the time required for 50% release of the drug.

Lateral diffusion coefficient (*D_L_*) was calculated via the slope of the plot between ln(1 − *M_t_*/*M*_oo_) and time.

*D_L_* = (slope l^2^/8) (7)

### 2.7. Kinetics of Drug Release

The kinetics of drug release from the CH-Ag bionanocomposite was studied by finding the best fit on the kinetic models, such as zero order(Equation (8)), first order (Equation (9)), Higuchi model (Equation (10)), Korsmeyer–Peppas model (Equation (11)) and Hixson–Crowell model (Equation (12)) [34,35,36].

(8) $$\frac{M_{t}}{M_{\infty }}=k_{0}t$$

(9) $$\ln(1-\frac{M_{t}}{M_{\infty }})=-k_{1}t$$

(10) $$\frac{M_{t}}{M_{\infty }}=k_{2}t^{\frac{1}{2}}$$

(11) $$\frac{M_{t}}{M_{\infty }}=k_{3}t^{n}$$

(12) $$\sqrt[{3}]{Q_{o}}-\sqrt[{3}]{Q_{t}}=k_{4}t$$

In these equations, *t* is the drug release time, n represents the release exponent, and *Q_o_* and *Q_t_* are the amount of drug loaded and drug release at time t in the synthesized bionanocomposite, respectively. *k*_0_, *k*_1_, *k*_2_, *k*_3_ and *k*_4_ are rate constants of the drug release of different kinetics models.

## 3. Results and Discussion

### 3.1. Characterization

#### 3.1.1. TEM Analysis

**Figure 3:**
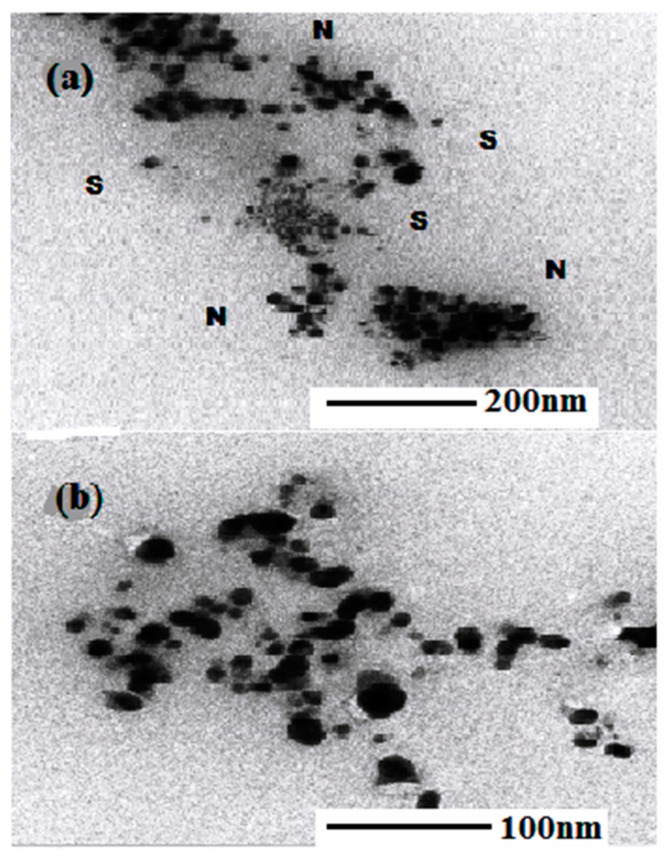
TEM images (100 nm scale bar) of AgNPs synthesized by *S. officinarum* extract: (**a**) AgNPs surrounded by stabilizing agents and (**b**) AgNPs.

The morphology and size of the synthesized AgNPs were studied using an JEOL-JEM-2100 transmission electron microscope (TEM). The TEM sample was prepared by placing the sample onto a carbon film, supported on a copper grid, followed by solvent evaporation under vacuum. The TEM images of AgNPs synthesized by *S. officinarum* are shown in (Figure 3a,b), which reveal the formation of AgNPs produced by *S. officinarum*. It was revealed, from the results, that there were stabilizing agents around the AgNPs (‘N’ depicted NPs and ‘S’ depicted the stabilizing agents). As was clear from Figure 3a,b, the presence of large sums of sucrose in *S. officinarum* might be accountable for the reduction and stabilizing of AgNPs.

#### 3.1.2. FTIR Analysis of Synthesized AgNPs

**Figure 4:**
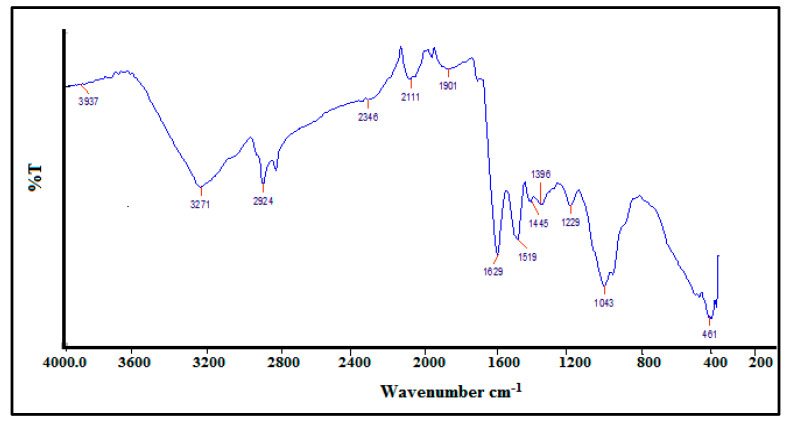
FTIR spectrums of AgNPs synthesized by *S. officinarum* extract.

The phytochemicals in the *S. officinarum* extract entailed in NPs synthesis were identified by FTIR. The FTIR spectrum of the synthesized AgNPs is shown in Figure 4. The peaks at 3937 cm^−1^ and 3271 cm^−1^ are due to the H-OH stretching of phenols. The reducing sugars of the *S. officinarum* extract might be acting as reducing agents to synthesize AgNPs. The adsorption peak at 1629 cm^−1^ is due to the C=O stretching of aldehydes and ketones. The peak at 1519 cm^−1^ corresponds to the N=O bending of nitro groups. The bands observed at 1446 cm^−1^ and 1396 cm^−1^ represent the C-O stretching of esters. The peaks at 1229, 1043 and 461 cm^−1^ correspond to the C-H stretching of alkenes.

#### 3.1.3. Visual Observation

**Figure 5:**
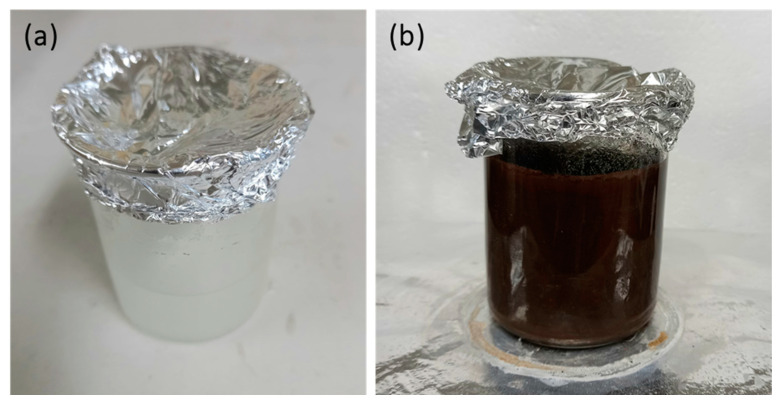
Visual examination of the synthesis of AgNPs: (**a**) silver nitrate solution without *S. officinarum* extract and (**b**) after the addition of *S. officinarum* extract to silver nitrate solution.

The production of NPs from plant extracts is gaining significant interest, due to the low production cost and eco-friendly nature. A color change is an indication of the synthesis of AgNPs using biological materials [34,35,36].After the addition of *S. officinarum* extract to the silver nitrate solution, there is a change in the color of the solution, which is an indication of the formation of AgNPs. The appearance of a brown color occurred within 5 min after the addition of *S. officinarum* extract, and the intensity of the brown color increased with time, and it attained a constant color after 12 h (Figure 5b). This change in the color was due to the excitation of surface plasmon resonance (SPR) of the synthesized AgNPs. Silver nitrate was treated as the control (Figure 5a). After 12 h, the change in colour from white to dark brown indicate the synthesized silver nanoparticles indicates the complete reduction of silver metal ions into silver nanoparticles. As a result of the excitation of surface plasmon resonance (SPR) of the silver nanoparticles, the color changed from white to brown.

**Figure 6:**
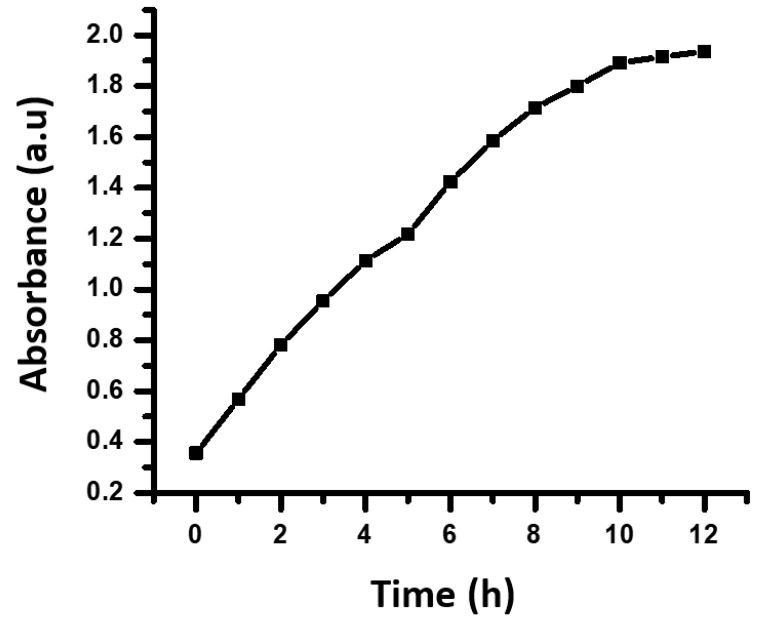
Absorbance of AgNPs synthesized by *S. officinarum* extracts at different time intervals.

The time-dependent visible spectra of AgNPs with *S. officinarum* aqueous leaf extract are shown in Figure 6. The visible spectra of AgNPs were recorded at different time intervals, from 0 h (initiation of reaction) to 12 h (Table 1). It was clear from the absorption results that after the addition of *S. officinarum* extract to silver nitrate, the intensity of the peak gradually increased with an increase in the reaction time from 0 to 12 h, which indicated that there was an increase in the concentration of silver nanoparticles in the reaction mixture with the passage of time.

### 3.2. Drug Loading and Release Studies

The drug entrapment efficiency of the synthesized bionanocomposite depends upon the physical and chemical nature and interaction of the drug and synthesized bionanocomposite. In this research work, itraconazole was used as a model drug. The results of drug entrapment efficiency in varied media are represented in Table 2. The higher EE of drugs was found in pH 7, followed by alkaline and acidic media. The results showed that synthesized bionanocomposite is very efficient for the entrapment of the drug, and can be utilized as a device for drug delivery [37,38].

**Figure 7:**
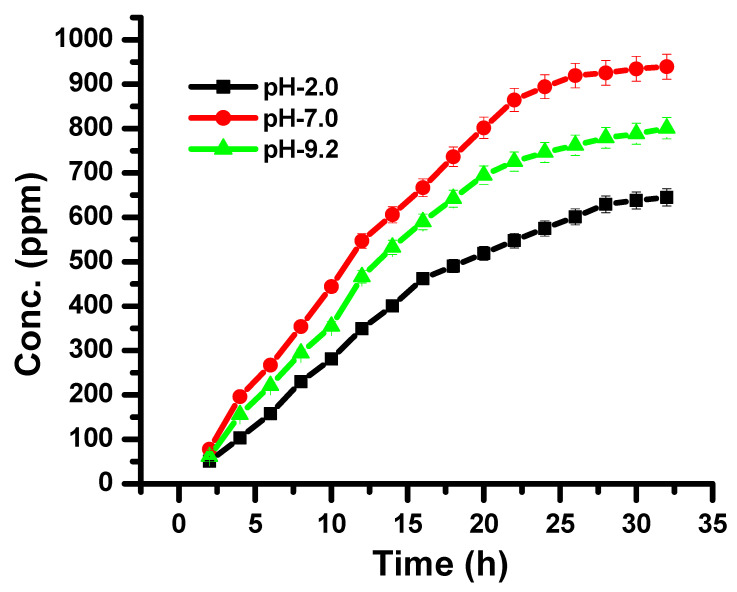
Effect of pH on release of itraconazole from CH-Ag bionanocomposite at different time intervals.

In vitro drug release, through synthesized CH-Ag bionanocomposite, was studied at pH 2, 7 and 9, as they provide the same physiological conditions as the gastric, blood stream and intestine, respectively. The drug release behavior through the synthesized CH-Ag bionanocomposite is given in Figure 7, which shows that the maximum drug release was found at pH 7 (93%), followed by pH 9.2 (79%), and the minimum was found at pH 2 (61%) (Figure 7) [39,40,41].The release behavior of the drug from the synthesized bionanocomposite was sustained release, without any burst. The interactions between the bionanocomposite and drug molecules lead to the prolonged and sustained release of the drug [42,43,44].

As the synthesized bionanocomposite matrix comes into contact with the aqueous solution, it starts to achieve a swollen state, where diffusion of the drug molecules takes place from the porous network of the CH-Ag bionanocomposite to the outer medium. Drug release from the CH-Ag bionanocomposite takes place by a diffusion mechanism. The values n and k for the release of itraconazole from the CH-Ag bionanocomposite were taken from the slope and intercept of the plot of M_t/_M_∞_ versus t in different pH conditions. The results of itraconazole clearly depicted that the drug release from the CH-Ag bionanocomposite followed a non-Fickian diffusion mechanism at pH 7, while it showed a Fickian mechanism in acidic and alkaline medium [44,45]. Drug release in a Fickian diffusion mechanism is controlled by concentration gradient-driven diffusion, and in the case of a non-Fickian diffusion mechanism, it is driven by polymer relaxation. Di were found to be higher than D_L_ (Table 3). The diffusion coefficient was higher at pH 7, demonstrating that drug diffusion was higher at a neutral pH, due to higher swelling in the network of the CH-Ag bionanocomposite at a neutral pH, followed by an alkaline and an acidic pH, respectively.

**Figure 8:**
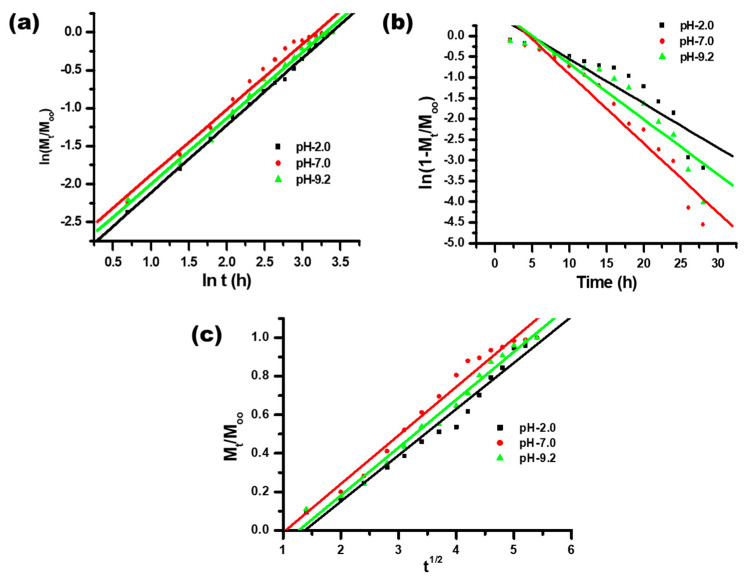
Plot of (**a**) ln (M_t_/M_∞_) vs. ln t; (**b**) ln(1 − M_t_/M_∞_) vs. time and (**c**) M_t_/M_∞_ vs. t^1/2^ for the release of itraconazole from synthesized CH-Ag bionanocomposite.

**Figure 9:**
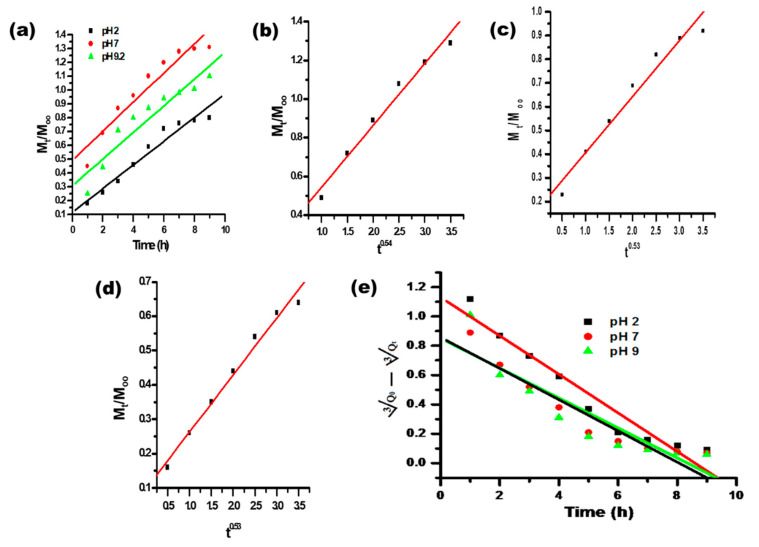
(**a**) Plot of M_t_/M_∞_ vs. time; (**b**) plots of M_t_/M_∞_ vs. t^n^ (n = 0.54) at pH 7; (**c**) plots of M_t_/M_∞_ vs. t^n^ (n = 0.53) at pH 2; (**d**) plots of M_t_/M_∞_ vs. t^n^ (n = 0.53) at pH 9.2; and (**e**) plot of root cube of √Q_o_ − √Q_t_ vs. time for the release of itraconazole from the synthesized CH-Ag bionanocomposite.

Varied kinetic models were used to study the in vitro drug release behavior. Different kinetics equations (8–12) were used for the linear plotting of drug release, as shown in Figure 8a–c and Figure 9a–e, producing the values of regression coefficient and rate constant. Drug release models were assessed from the values of the regression coefficient of the linear relationship between the releases of the drug at different times. The drug release results of different kinetic models are summarized in Table 4. The drug release profile corresponded best with the first-order kinetic model, having the highest regression coefficient value (Table 4). The first-order kinetic model of drug release from the synthesized CH-Ag bionanocomposite depicted a time-dependent profile, where the drug release rate constant depended upon the initial concentration of the drug present in the bionanocomposite [45,46].

### 3.3. Antibacterial Activity of AgNPs and CH-Ag Bionanocomposite

**Figure 10:**
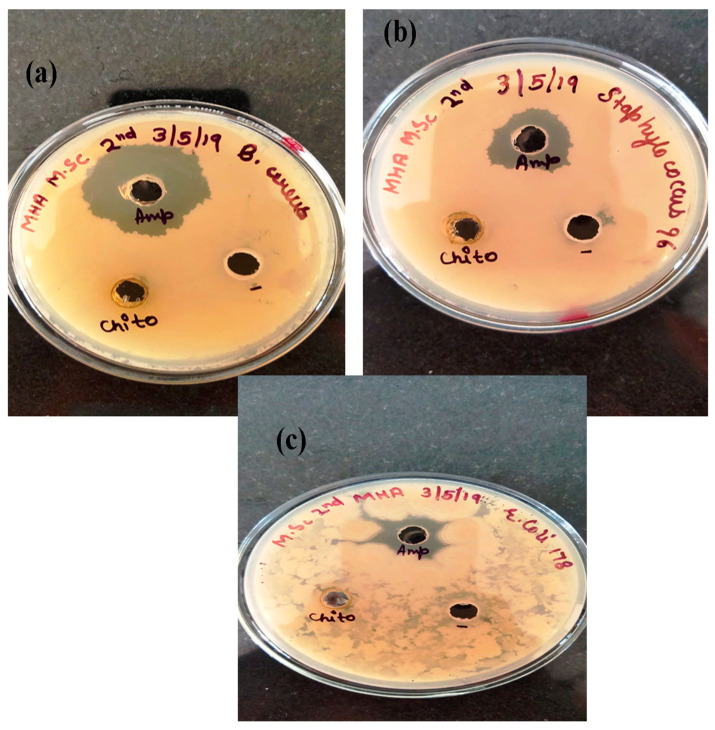
Antibacterial activity of NPs and CH-Ag bionanocomposite against (**a**) *Bacillus cereus*, (**b**)*Staphylococcus*, and (**c**) *Escherichia coli* by agar well diffusion method.

Bacterial infections are always a concern for the researcher, and extensive research is ongoing to find new antimicrobial drugs isolated from different sources. The present work utilized the synthesized AgNPs and CH-Ag bionanocomposite as an alternative to the antibacterial drug, as they have the capacity to be used against resistant bacteria [47,48]. The antibacterial activity of AgNPs and CH-Ag bionanocomposite was analyzed through the agar well diffusion method against *Bacillus cereus*, *Staphylococcus*, and *Escherichiacoli*. Erythromycin and ampicillin are antibiotics, which were used as the control. A single colony of bacterial strain was grown in a nutrient broth and incubated at 37 °C for 24 h. After incubation, each bacterial strain was spread onto the sterile agar plates. Then, the CH-Ag bionanocomposite (50 ppm) was poured in the well and the plates were incubated again at 37 °C for 24 h. After incubation, the zone of inhibition was measured. The zone of inhibition was found to be 10 mm, 11 mm, 11 mm and 9 mm in *Bacillus cereus*, *Staphylococcus*, and *Escherichiacoli*, respectively (Figure 10a–c). Thus, it is clear from the results that synthesized AgNPs and CH-Ag bionanocomposite are potentially very effective against different strains of bacteria [49,50].

## 4. Conclusions

An environmentally friendly method was used to synthesize the silver nanoparticles using *Saccharum officinarum* plant extracts, and then form CH-Ag bionanocomposite. The AgNPs were found to have a size between 50 and 100 nm. The synthesized CH-Ag bionanocomposite was then evaluated as a tool to determine the release profile of drugs, such as itraconazole, at different pH levels (pH 2, 7, and 9). It was clear from the results that drug release from the CH-Ag bionanocomposite occurred in a sustained manner. The maximum drug release occurred at pH 7, followed by pH 9.2, and the minimum drug release occurred at pH 2. The drug release followed a non-Fickian diffusion mechanism at neutral pH, and a Fickian diffusion mechanism at pH 2 and 9.2. The release profiles of the drug were best fitted in the first-order kinetic model. The synthesized CH-Ag bionanocomposite has been shown to be effective against different bacterial strains, including *Bacillus cereus*, *Staphylococcus*, and *Escherichia coli*. Hence, from the foregoing discussion, it can be concluded that the synthesized CH-Ag bionanocomposite could be a potential drug delivery device with controlled and site-specific delivery.

## Figures and Tables

**Table 1:** Visual observation of AgNPs with time.

Time (h)	Absorbance
0	0.356
1	0.569
2	0.781
3	0.954
4	1.114
5	1.217
6	1.423
7	1.586
8	1.715
9	1.8
10	1.892
11	1.915
12	1.937

**Table 2:** Itraconazole drug entrapment efficiency onto synthesized CH-Ag bionanocomposite at different pH.

Drugs	pH 2	pH 7	pH 9.2
	Drug Entrapment Efficiency (EE) %		
Itraconazole	64.12 ± 0.10	94.56 ± 0.11	75.03 ± 0.09

**Table 3:** Diffusion exponent ‘n’, gel characteristic constant ‘k’and diffusion coefficient of itraconazole drug release through CH-Ag bionanocomposite at different pH.

Drug	pH	Drug Release (mg) Itraconazole		‘n’	‘k’	Diffusion Coefficient (cm^2^h^−1^)			Mechanism
		Initial	Final			D_i_	D_A_	D_L_	
**Itraconazole**	**2**	**0.75**	**9.2**	0.462	1.431	0.176	0.021	0.08	Fickian
	**7**	**1.14**	**14.3**	1.064	1.256	0.329	0.189	0.094	Non-Fickian
	**9.2**	**0.89**	**11.45**	0.492	1.123	0.297	0.079	0.091	Fickian

**Table 4:** Kinetic interpretation of different models for the release of itraconazole from CH-Ag bionanocomposite.

Drug	Kinetic Model		pH 2	pH 7	pH 9.2
**Itraconazole**	**Zero order**	**R^2^**	0.769	0.929	0.842
		**k_0_ (mg h^−1^)**	0.102	0.181	0.135
	**First order**	**R^2^**	0.994	0.999	0.997
		**k_1_ (h^−1^)**	0.163	0.192	0.187
	**Higuchi**	**R^2^**	0.936	0.954	0.949
		**k_2_ (h^−1^^/2^)**	0.489	0.672	0.598
	**Korsmeyer–Peppas**	**R^2^**	0.967	0.993	0.982
		**k_3_ (h^−n^)**	0.479	0.596	0.501
	**Hixson–Crowell**	**R^2^**	0.714	0.798	0.752
		**k_4_ (h^−1^^/3^)**	0.132	0.167	0.149

## Data Availability

The data presented in this study are available on request from the corresponding authors.

## References

[1] Matteis V.D., Rizzello L., Ingrosso C., Liatsi-Douvitsa E. (2019). Cultivar-Dependent Anticancer and Antibacterial Properties of Silver Nanoparticles Synthesized Using Leaves of DifferentEuropaea Trees. Nanomaterials.

[2] Naushad M., ALOthman Z.A. (2015). Separation of toxic Pb^2+^ metal from aqueous solution using strongly acidic cation-exchange resin: Analytical applications for the removal of metal ions from pharmaceutical formulation. Desalin. Water Treat..

[3] Saruchi, Thakur P., Kumar V. (2019). Kinetics and thermodynamic studies for removal of methylene blue dye by biosynthesize copperoxide nanoparticles and its antibacterial activity. J. Environ. Health Sci. Eng..

[4] Sethi S., Kaith B.S., Saruchi K.V. (2019). Fabrication and characterization of microwave assisted carboxymethyl cellulose-gelatin silver nanoparticles imbibed hydrogel: Its evaluation as dye degradation. React. Funct. Polym..

[5] Naushad M. (2014). Surfactant assisted nano-composite cation exchanger: Development, characterization and applications for the removal of toxic Pb^2+^ from aqueous medium. Chem. Eng. J..

[6] Saruchi, Kumar V. (2020). Effective degradation of rhodamine B and congo red dyes over biosynthesized silver nanoparticles-imbibed carboxymethylcellulose hydrogel. Polym. Bull..

[7] Ahmad M.B., Lim J.J., Shameli K., Ibrahim N.A., Tay M.Y. (2011). Synthesis of Silver Nanoparticles in Chitosan, Gelatin and Chitosan/Gelatin Bionanocomposites by a Chemical Reducing Agent and Their Characterization. Molecules.

[8] Balasubramani K., Sivarajasekar N., Naushad M. (2020). Effective adsorption of antidiabetic pharmaceutical (metformin) from aqueous medium using graphene oxide nanoparticles: Equilibrium and statistical modelling. J. Mol. Liq..

[9] Hwi J.K., Lestner J.J., Hope W.W. (2013). Itraconazole: Recent update on pharmacology and clinical use for treatment of invasive and allergic fungal infections. Expert Opin. Drug Metab. Toxicol..

[10] Kumar P., Gnanajobitha G., Vanaja M., Pavunraj M., Annadurai G. (2017). Green synthesis of silver nanoparticles and silver based chitosan bionanocomposite using stem extract of sugar cane and assessment of its antibacterial activity. Adv. Nat. Sci. Nanosci. Nanotechnol..

[11] Kaith B.S., Jindal R., Kapur G.S. (2014). Synthesis of acrylic acid based hydrogel: Its evaluation for controlled release of antiulcerative drug pantoprazole sodium. J. Chin. Adv. Mater. Soc..

[12] Naushad M., Sharma G., Alothman Z.A. (2019). Photodegradation of toxic dye using Gum Arabic-crosslinked-poly(acrylamide)/Ni(OH)_2_/FeOOH nanocomposites hydrogel. J. Clean. Prod..

[13] Naushad M., Mittal A., Rathore M., Gupta V. (2015). Ion-exchange kinetic studies for Cd(II), Co(II), Cu(II), and Pb(II) metal ions over a composite cation exchanger. Desalin. Water Treat..

[14] Abdelhamid H.N., El-Ber H.M., Metwally A.A., Elshazl M., Hathout R.M. (2019). Synthesis of CdS-modified chitosan quantum dots for the drug delivery of Sesamol. Carbohdrate Polym..

[15] Bhumkar D.R., Joshi H.M., Sastry M., Pokharkar V.B. (2007). Chitosan reduced gold nanoparticles as novel carriers for transmucosal delivery of insulin. Pharm. Res..

[16] Bagherzade G., Tavakoli M.M., Namaei M.H. (2017). Green synthesis of silver Nanoparticles using aqueous extract of saffron (*Crocussativus* L.) wastages and its antibacterial activity against six bacteria. Asian Pac. J. Trop. Biomed..

[17] Gopinath V., Priyadarshini S., Loke M.F., Kumar J.K., Marsili E., Ali D.M., Velusamy P., Vadivelu J. (2017). Biogenic synthesis, characterization of antibacterial silver nanoparticles and its cell cytotoxicity. Arab. J. Chem..

[18] Li S., Shen Y., Xie A., Yu X., Qiu L., Zhang L., Zhang Q. (2007). Green synthesis of silver nanoparticles using *Capsicum annuum* L. extract. Green Chem..

[19] Roy A. (2017). Synthesis of silver nanoparticles and its biological application: A review. Res. Rev. Biosci..

[20] Sharma G., Pathania D., Naushad N., Kothiyal N.C. (2014). Fabrication, characterization and antimicrobial activity of polyaniline Th (IV) tungstomolybdophosphate nanocomposite material: Efficient removal of toxic metal ions from water. Chem. Eng. J..

[21] Hanna D.H., Saad G.R. (2019). Encapsulation of ciprofloxacin with in modified xanthan gum-chitosan based hydrogel for drug delivery. Bioorganic Chem..

[22] Wang Y., Wanga W.W., Wanga W.A. (2013). Efficient adsorption of methylene blue on an alginate-based nanocomposite hydrogel enhanced by organo-illite/smectiteclay. Chem. Eng. J..

[23] Sethi S., Kaith B.S., Kaur M., Sharma N., Khullar S. (2019). Study of a Cross-Linked Hydrogel of Karaya Gum and Starch as a Controlled Drug Delivery System. J. Biomater. Sci..

[24] Saruchi, Sharma M., Hatshan M.R., Kumar V., Rana A. (2021). Sequestration of Eosin Dye by Magnesium (II)-Doped Zinc Oxide Nanoparticles: Its Kinetic, Isotherm, and Thermodynamic Studies. J. Chem. Eng. Data..

[25] Shafaghi S., Moghadam P.M., Fareghi A.R., Baradarani M.M. (2014). Synthesis and characterization of a drug-delivery system based on melamine-modified poly(vinylacetate-*co*-maleicanhydride) hydrogel. J. Appl. Sci..

[26] Prabaharan M. (2008). Review paper: Chitosan derivatives as promising materials for controlled drug delivery. J. Biomter Appl..

[27] Sharma G., Naushad N., Pathania D., Mittal A., Eldesoky G.E. (2015). Modification of Hibiscus cannabinus fiber by graft copolymerization: Application for dye removal, Desalin. Water Treat..

[28] Keller B.L., Lohmann C.A., Kyeremateng S.O., Fricker G. (2022). Synthesis and Characterization of Biodegradable Poly(butylcyanoacrylate) for Drug Delivery Applications. Polymers.

[29] Srikhao N., Kasemisiri P., Lorwanishpaisam N., Okhawilai M. (2021). Green synthesis of silvern anoparticles using sugarcane leaves extract for colorimetric detection of ammonia and hydrogenperoxide. Res. Chem. Intermed..

[30] Sethi S., Saruchi K.B.S., Kaur M., Sharma N., Kumar V. (2020). Cross-linked xanthan gum–starch hydrogels as promising materials for controlled drug delivery. Cellulose.

[31] Kaith B.S., Saruchi J.R., Bhatti M.S. (2012). Screening and RSM optimization for synthesis of a gum tragacanth–acrylic acid based device for in situ controlled cetirizine dihydrochloride release. Soft Matter.

[32] Zhou H.Y., Zhang Y.P., Zhang W.F., Chen X.G. (2011). Biocompatibility and characteristics of injectable chitosan-based thermosensitive hydrogel for drug delivery. Carbohydr. Polym..

[33] Saruchi, Kaith B.S., Jindal R., Kumar V., Bhatti M.S. (2014). Optimal response surface design of Gum tragacanth-based poly[(acrylicacid)-coacrylamide ]IPN hydrogel for the controlled release of the antihypertensive drug losartan potassium. RSC Adv..

[34] Singh B., Kumar A. (2018). Network formation of MoringaOleifera gum by radiation induced crosslinking:Evaluation of drug delivery, network parameters and biomedical parameters. Int. J. Biol. Macromol..

[35] Singh B., Sharma V. (2014). Influence of polyme r network parameters of tragacanth gum-based pH responsive hydrogels on drugdelivery. Carbohydr. Polym..

[36] Singh B., Sharma V. (2017). Crosslinking of poly(vinylpyrrolidone)/acrylic acid with tragacanth gum for hydrogels formation for use in drug delivery applications. Carbohydr. Polym..

[37] Treesuppharata P., Rojanapanthua C., Siangsanohb H., Manuspiyac S. (2017). Synthesis and characterization of bacterial cellulose and gelatin-based hydrogel composites for drug-delivery systems. Biotechnol. Rep..

[38] Wei W., Li J., Qi X., Zhong Y., Zuo G., Su T., Zhang J. (2017). Synthesis and characterization of a multisensitive polysaccharides hydrogel for drug delivery. Carbohydr. Polym..

[39] Liu Y., Sui Y., Liu C., Liu C., Wu M., Li B., Li Y. (2018). A physically crosslinked polydopamine/nanocellulose hydrogel as potential versatile vehicles for drug delivery and wound healing. Carbohydr. Polym..

[40] Shakeel A., Mudasir A. (2016). A review on plant extract mediated synthesized of silver nanoparticles for antimicrobial application: A green expertise. J. Adv. Res..

[41] Weitz I.S., Maoz M., Panitz D., Eichler S., Segal E. (2015). Combination of CuO nanoparticles and fluconazole: Preparation, characterization, and antifungal activity against Candida albicans. J. Nanopart. Res..

[42] Wan G., Ruan L., Yin Y., Yang T., Ge M., Cheng X. (2016). Effect of Silver Nanoparticles in combination with antibiotiotics on the resistant bacteria Acinetobacterbaumannii. Int. J. Nanomed..

[43] Usman M.S., Zowalaty M.E.E., Shameli K., Zainuddin N., Salama M., Ibrahim N.A. (2013). Synthesis, characterization and antimicrobial properties of copper nanoparticles. Int. J. Nanomed..

[44] Tiwari D.K., Behari J., Sen P. (2008). Time and dose-dependent antimicrobial potential of Ag nanoparticles synthesized by top-down approach. Curr. Sci..

[45] Thati V., Roy A.S., Prasad A.N., Shivannavar C.T., Gaddad S.M. (2010). Nanostructured zinc oxide enhances the activity of antibiotics against Staphylococcus aureus. J. Biosci. Tech..

[46] Sanpui P.A., Murugadoss P., Prasad S., Ghosh A., Chattopadhyay B. (2008). The antibacterial properties of a novel chitosan-Ag-nanoparticle composite. Int. J. Food Microbiol..

[47] Ezzat H., Rady M., Hathout M.R., Abdel-Halim M., Mansour S. (2021). Enhanced anti-bacterial effect of kojic acid using gelatinized coreliposomes: A potential approach to combat antibiotic resistance. J. Drug Deliv. Sci. Technol..

[48] Abdelhamid S.M., El-Hasseiny L.S. (2017). Combined efficacy of thymol and silver nanoparticle against *Staphylococcus Aureus*. Afr. J. Microbiol. Res..

[49] Rozhin A., Batasheva S., Kruychkova M., Cherednichenko Y., Rozhina E., Fakhrullin R. (2021). Biogenic silver nanoparticles: Synthesis and application as antibacterial and antifungal agents. Micromachines.

[50] Singh M., Kumar M., Kalaivani R., Kumaraguru A.K. (2013). Metallic silver nanoparticle: A therapeutic agent in combination with antifungal drug against human fungal pathogen. Bioprocess Biosyst Eng..

